# The evaluation of low-density lipoprotein cholesterol goals achieved in patients with established cardiovascular disease and/or hyperlipidaemia receiving lipid-lowering therapy: the South African Not At Goal study (SA-NAG)

**Published:** 2008-04

**Authors:** Akash Ramjeeth, Neil Butkow, Frederick Raal, Mandisa Maholwana-Mokgatlhe

**Affiliations:** Department of Pharmacy and Pharmacology, School of Therapeutic Science, University of the Witwatersrand, Johannesburg; Department of Pharmacy and Pharmacology, School of Therapeutic Science, University of the Witwatersrand, Johannesburg; Department of Medicine, Division of Endocrinology and Metabolism, University of the Witwatersrand, Johannesburg; Merck Sharp & Dohme (MSD), Halfway House, Midrand

## Abstract

**Aim:**

Cardiovascular disease (CVD) is the leading cause of morbidity and mortality worldwide. Dyslipidaemia is a major risk factor that leads to the clinical sequelae of CVD. As a result, it has become essential for South Africa to update its guidelines for the management of dyslipidaemia, and the South African scientific community has recently adopted the European guidelines on CVD prevention in clinical practice. The South African Not at Goal study (SA-NAG) was a survey done to determine the percentage of patients on lipid-lowering therapy who were not achieving guideline-specified low-density lipoprotein cholesterol (LDL-C) goals.

**Methods:**

In this cross-sectional study, dyslipidaemic and/or CVD patients on lipid-lowering therapy for more than four months were enrolled. Volunteers had their demographic data and previous medical history documented. Blood samples from these patients were analysed (using standardised methods) to obtain fasting blood lipid and glucose levels.

**Results:**

In total, 1 201 patients (age 58 ± 11.4 years) were recruited by physicians and general practitioners from across South Africa. Under the new guidelines, 41% of patients were defined as low risk (LR) and 59% were high risk (HR). Sixty-three per cent of LR patients and 77% of HR patients (71% overall) did not achieve their LDL-C target goals of 2.5 and 3.0 mmol/l, respectively. The LR and HR patients who did not achieve their LDL-C goals were on average 19% (0.7 mmol/l ± 0.5) and 31% (1.1 mmol/l ± 1.1) above their LDL-C target levels, respectively.

**Conclusions:**

These results suggest that a considerable number of patients fall into the category ‘not at goal’ LDL-C. Patients who failed to achieve goal were also far above their LDL-C target levels. The adoption of the new guidelines will necessitate enhanced disease management to reduce the disease burden.

## Summary

Cholesterol reduction decreases the risk of all major coronary events, coronary revascularisation and stroke.^1^ This link to cardiovascular disease (CVD) is further supported by recent trials demonstrating improved outcomes and reduced progression of coronary atherosclerosis with intensive low-density lipoprotein cholesterol (LDL-C) lowering.^2^-^6^ The benefit of lowering LDL-C is indisputable and its association with atherosclerosis is now beyond doubt. In South Africa, CVD primarily affects the white and Indian populations in the form of ischaemic heart disease.^7^ At present, coronary heart disease rates among the black population are lower in comparison, and stroke is the major manifestation of CVD.^7^-^9^ However, South Africa is a developing nation and with increases in urbanisation, it is expected that CVD rates and risk factors among the population will increase.^7^

Many countries have published guidelines to govern the optimal management of CVD. Guidelines are modelled on the most current evidence-based medicine at the time of their publication. 10 At the time of this survey (2005−2006), South Africa followed a set of lipid guidelines published in February 2000.^11^ These guidelines are arguably outdated in terms of the most recent evidence available. Taking into account the most current scientific evidence, the European Society of Cardiology (ESC) guidelines have been adopted to align South African clinical practice with international standards (July 2006).^12^-^16^

Under these guidelines, assessment of CVD risk using risk charts and the treatment of dyslipidaemia as the primary target of therapy are supported. Goals of treatment are separated into two categories, low-risk (LR) and high-risk (HR) patients. The cholesterol goals for treatment in HR patients (Table 1) are lower than those of the previous South African guidelines. The United States and United Kingdom societies advise even lower LDL-C goals of < 1.8 mmol/l (70 mg/dl) in patients at very high risk for CVD, as recent evidence from intensive lipid-lowering trials supports these lower goals.^3^-^6^,^17^,^18^ The updated guidelines also support the clinical significance of the metabolic syndrome (MS) for predicting CVD events, and underline lifestyle changes, principally reducing body weight and increasing physical activity, as treatment priorities in these patients.

**Table 1.** Guideline Cholesterol Goals For Treatment [SA (ESC) Guidelines 2006]

	*Goal of treatment*	
*Risk Category*	*TC*	*LDL-C*
Low risk	< 5.0 mmol/l (190 mg/dl)	< 3.0 mmol/l (115 mg/dl)
High risk	< 4.5 mmol/l (175 mg/dl)	< 2.5 mmol/l (100 mg/dl)

TC: total cholesterol; LDL-C: low-density lipoprotein cholesterol.

Even though reductions in risk can be obtained by lifestyle changes and drug therapy, numerous surveys worldwide have shown great disparity between guideline goals of treatment and clinical practice.^19^-^23^ A more troubling statistic from these surveys is that patients at highest risk of suffering a CVD event have the lowest rate of achieving lifestyle, risk factor and therapeutic targets.^24^

Relatively little is known about the extent to which patients are able to reach LDL-C target levels in South Africa. Available evidence demonstrates the lack of appropriate management in an environment of increasing disease prevalence and CVD risk factors.^26^-^27^ In order to reduce mortality and morbidity due to vascular disease, as well as close the gap between guidelines and clinical practice, there is a need to evaluate the number of patients who are not at guideline-specified goals with current lipid-lowering therapy in South Africa. This survey therefore assessed: (1) the percentage of patients on lipid-lowering therapy who were not achieving guideline-specified LDL-C target levels and (2) the average percentage difference between the LDL-C levels that were being achieved and guideline goals (how far above goal level).

## Methods

Ethics approval for this research was obtained from the Human Research Ethics Committee of the University of the Witwatersrand, South Africa. This study was a multicentre, cross-sectional survey of randomly selected patients from private hospitals and private practices throughout South Africa. Volunteers were recruited from both general practitioners and physicians (investigators). Trained fieldworkers briefed investigators in study procedures. The study was designed as a single visit of the volunteer to their investigator. At this visit, the patient had a sample of blood drawn, which was sent to the laboratory for the determination of fasting blood lipid and glucose levels, and haemoglobin A_1c_ (HbA_1c_ − measured only in diabetic patients) levels. Patient demographic data and the patients’ past medical history were recorded and analysed in conjunction with laboratory results to formulate relevant conclusions about goal achievement.

Patients between the ages of 18 and 80 years old who were willing to sign an informed consent form, were on the same lipid-lowering therapy for at least four months, and satisfied the inclusion and exclusion criteria, were recruited into the study. Patients who had type 1 or type 2 diabetes with HbA_1c_ > 20% were excluded. Patients were also excluded from the study if they had had trauma, recent surgery that required anaesthesia or a myocardial infarction within the 12 weeks before enrolment. Patients who had an acute infection that required current antibiotic therapy, those who had an abrupt change to their diets in the one month preceding the study and women who were pregnant, breastfeeding and/or six months or less *post partum* were also ineligible for the study.

Patient case report forms were designed to record demographic information regarding age, gender, race, waist circumference, height and weight. Body mass index (BMI) was calculated as: mass (kg)/[height (m)]^2^. Obesity was defined as a BMI of ≥ 30 kg/m^2^, while overweight patients were classified according to a BMI of > 25 but < 29 kg/m^2^. Other information recorded was the patients’ cardiovascular risk factors: CVD history (defined by clinical diagnosis of angina, stroke, myocardial infarction, peripheral vascular disease), family history of CVD (familial hypercholesterolaemia and family history of coronary artery disease before 55 years of age in male first-degree relatives, or before 65 years of age in female first-degree relatives), blood pressure (BP) on the day of the investigation and whether the patient had a history of hypertension (physician-diagnosed or on an antihypertensive medication), smoking, exercise or diet therapies, diabetes (physician-diagnosed or on an antidiabetic medication), if the patient was female, the result of urine pregnancy test, and the results of blood analysis (total cholesterol, LDL-C, triglycerides, HDL-C, blood glucose and HbA_1c_). All medications utilised and doses were recorded.

The updated South African guidelines (adopted ESC − released July 2006) were used to stratify patients into the two risk categories.^13^ Patients who fell into the low-risk category were the healthy asymptomatic patients with a 10-year risk of a major coronary event of < 20%. Patients in the high-risk category included those patients with established CVD, type 2 diabetes, severe genetic lipid disorders, patients with a 10-year risk of a major coronary event of ≥ 20% now or extrapolated to age 60, or markedly raised levels of single risk factors [total cholesterol levels of ≥ 7.5 mmol/l (290 mg/dl), LDL-C levels of ≥ 6 mmol/l (230 mg/dl), BP of ≥ 180/110 mmHg]. Risk calculations for 10-year major coronary events were performed using the Framingham risk charts and the criteria used for diagnosis of the MS were those set forth by the American Heart Association/National Heart, Lung and Blood Institute (AHA/NHLBI) scientific statement published in 2005.^15^,^16^,^28^

All laboratory determinations were performed at a central laboratory [Bio-Analytical Research Corporation South Africa Pty Ltd (BARC), a division of Lancet Laboratories, Clinical Trials Laboratory, Richmond, Johannesburg], which was a South African National Accreditation System (SANAS)-accredited laboratory. Fasting blood samples were drawn from volunteers while seated or lying down and couriered to the central laboratory within 24 hours of collection. All lipid (except LDL-C) and blood glucose determinations were made using an Abbot Laboratories Aeroset® c8000 system. Low-density lipoprotein cholesterol levels were determined indirectly using the Friedewald equation.^29^ Haemoglobin A_1c_ measurements were made using a Bio-Rad Variant™ II haemoglobin testing system. All assays and reagents were standardised according to the manufacturer’s recommendations. Patients with triglyceride levels of > 10.34 mmol/l (915 mg/dl) were not included in the statistical analysis because excessive elevations in triglycerides affect Friedewald equation LDL-C measurements.

## Statistical analysis

The primary study end-point was rate of failure, which is the percentage of patients who did not achieve South African guideline (ESC)-specified LDL-C target levels. Data were analysed using databases, frequency distributions and descriptive statistics between all risk factors and therapies. Once the database was complete, missing fields and fields that were biologically improbable or in conflict with the entry criteria were queried and corrected, or the patient was removed from the study. Statistical analyses were carried out using GraphPad InStat™ V2.05. Data are presented as means (± standard deviation). The Turkey-Kramer multiple-comparisons test was used for comparing differences between groups, and a probability level of < 0.05 was considered significant.

## Results

The survey was conducted from November 2005 to November 2006. A total of 1 345 volunteers were enrolled in the survey from 29 sites. Of the 1 345 patients, 144 did not meet the inclusion or exclusion criteria. Among the most common reasons for exclusion were an elevated HbA_1c_ > 10% or inclusion of subjects not on lipid-lowering therapy. The data for the remaining 1 201 evaluable patients (44% female) were analysed in this report. Of these patients, 986 (82%) were white, 126 (10.5%) were Indian, 73 (6.1%) were coloured and eight (0.7%) were black. The mean (± SD) age of patients was 58.3 (± 11.4) years.

## Lipid goals

**Fig. 1.**
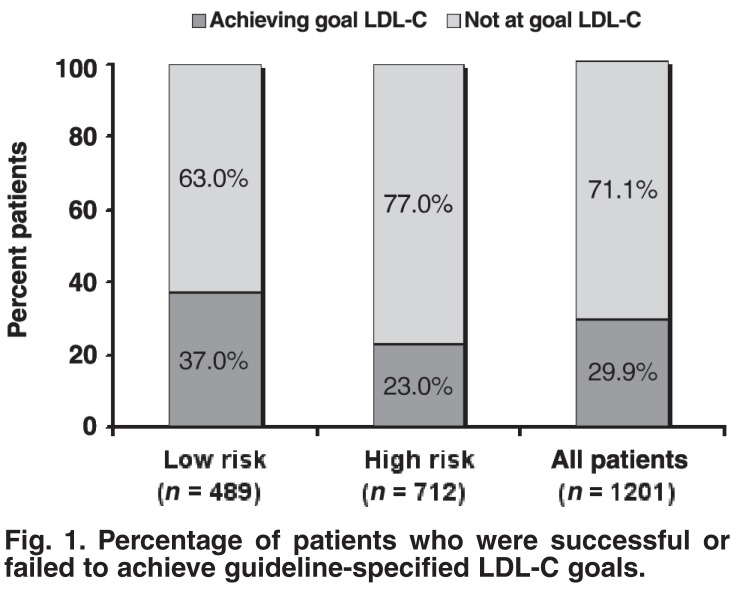
Percentage of patients who were successful or failed to achieve guideline-specified LDL-C goals.

The 1 201 patients included in analysis were divided into the LR and HR categories as described earlier. Of these, 489 (40.7%) and 712 (59.3%) were classified as LR and HR, respectively. In the LR group, 63% (306) of patients, compared to 77% (548) of patients in the HR group did not achieve their guideline-specified LDL-C levels (Fig. 1). Overall, 71% (854) of all participating patients were not at goal LDL-C levels. The mean (± SD) LDL-C for those LR and HR patients achieving target level was 2.5 (± 0.4) and 2.1 (± 0.3) mmol/l (81 and 100 mg/dl), respectively. The mean (± SD) LDL-C levels of those patients not attaining goal were 3.7 (± 0.5) mmol/l and 3.6 (± 1.1) mmol/l (143 and 139 mg/dl) in the LR and HR groups, respectively.

**Fig. 2.**
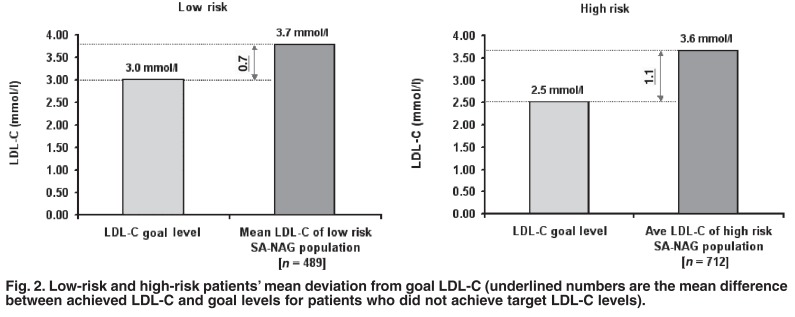
Low-risk and high-risk patients’ mean deviation from goal LDL-C (underlined numbers are the mean difference between achieved LDL-C and goal levels for patients who did not achieve target LDL-C levels).

In all age groups, mean LDL-C of patients not achieving goal was significantly greater than goal LDL-C levels across the LR and HR categories (*p* < 0.05). The mean difference between recommended LDL-C goal levels and the current population mean for patients not at goal was 0.7 (± 0.5) and 1.1 (± 1.1) mmol/l (19 and 31%) away from goal for the LR and HR groups, respectively (Fig. 2). Of the patients not at goal, 60% (496) of patients had LDL-C levels of > 18% away from their individual SA (ESC) guideline goal levels. The mean total cholesterol level for both the LR and HR patients not achieving goal LDL-C levels was 5.9 mmol/l (228 mg/dl). Overall, 73% (881) of patients did not achieve total cholesterol goals (Table 2).

**Table 2.** Lipid Risk Factor Mean And Frequency Table For Female And Male Patients

*Risk group*	*Low risk*					*High risk*				
*Age group (years)*	*≤ 49*	*50−59*	*60−69*	*≥ 70*	*overall*	*≤ 49*	*50−59*	*60−69*	*≥ 70*	*overall*
*Gender*	*Female (n = 329)*					*Female (n = 204)*				
	*n = 41*	*n = 115*	*n = 113*	*n = 61*	*n = 329*	*n = 46*	*n = 49*	*n = 63*	*n = 46*	*n = 204*
Total cholesterol, mean (± SD)	5.7 (0.8)	5.6 (0.9)	5.6 (0.8)	5.4 (0.7)	5.6 (0.8)	6.3 (2.3)	5.6 (1.6)	5.7 (1.3)	5.4 (1.0)	5.7 (1.6)
LDL cholesterol, mean (± SD)	3.5 (0.9)	3.3 (0.8)	3.4 (0.8)	3.1 (0.6)	3.3 (0.8)	4.1 (2.2)	3.3 (1.3)	3.4 (1.1)	3.1 (0.8)	3.5 (1.5)
HDL cholesterol, mean (± SD)	1.5 (0.4)	1.6 (0.5)	1.6 (0.3)	1.6 (0.4)	1.6 (0.4)	1.4 (0.4)	1.5 (0.4)	1.5 (0.4)	1.5 (0.4)	1.5 (0.4)
Triglycerides, mean (± SD)	1.5 (0.8)	1.5 (0.9)	1.6 (0.9)	1.5 (0.7)	1.5 (0.8)	1.7 (1.1)	2.0 (1.3)	1.7 (0.8)	1.7 (1.2)	1.8 (1.1)
LDL NAG, no (%)	32 (78)	72 (63)	72 (64)	37 (61)	213 (65)	36 (78)	37 (76)	50 (79)	34 (74)	157 (77)
LDL NAG (mmol/l), mean (± SD)	3.8 (0.7)	3.8 (0.6)	3.8 (0.6)	3.5 (0.4)	3.7 (0.6)	4.6 (2.1)	3.7 (1.3)	3.7 (1)	3.4 (0.7)	3.9 (1.4)
mmol/l from goal (± SD)	0.8 (0.7)	0.8 (0.6)	0.8 (0.6)	0.5 (0.4)	0.7 (0.6)	2.1 (2.1)	1.2 (1.3)	1.2 (1.0)	0.9 (0.7)	1.4 (1.4)
% from goal	21	21	21	14	19	46	32	32	27	36
*Gender*	*Male (n = 160)*					*Male (n = 508)*				
	n = 5*	*n = 49*	*n = 83*	*n = 23*	*n = 160*	*n = 178*	*n = 134*	*n = 116*	*n = 80*	*n = 508*
Total cholesterol, mean (± SD)	4.9 (0.5)	5.3 (0.8)	5.3 (0.9)	5.2 (0.6)	5.3 (0.8)	5.9 (1.2)	5.5 (1.2)	4.9 (0.9)	4.6 (0.9)	5.3 (1.2)
LDL cholesterol, mean (± SD)	2.9 (0.7)	3.1 (0.8)	3.2 (0.8)	3.1 (0.6)	3.2 (0.7)	3.6 (1.2)	3.3 (1.5)	2.9 (0.7)	2.7 (0.7)	3.2 (1.0)
HDL cholesterol, mean (± SD)	1.1 (0.1)	1.5 (0.3)	1.4 (0.3)	1.5 (0.2)	1.4 (0.3)	1.2 (0.2)	1.2 (0.3)	1.2 (0.3)	1.3 (0.4)	1.2 (0.3)
Triglycerides, mean (± SD)	1.8 (1.1)	1.64 (1)	1.6 (1.1)	1.3 (0.5)	1.6 (1.0)	2.3 (1.5)	2.2 (1.4)	1.7 (0.9)	1.6 (0.8)	2.0 (1.3)
LDL NAG, no (%)	2 (40)	28 (57)	50 (60)	13 (57)	93 (58)	154 (87)	105 (78)	85 (73)	47 (59)	391 (77)
LDL NAG (mmol/l), mean (± SD)	3.5 (0.6)	3.7 (0.5)	3.7 (0.5)	3.5 (0.3)	3.7 (0.5)	3.9 (1.1)	3.6 (0.9)	3.2 (0.6)	3.1 (0.5)	3.6 (0.9)
mmol/l from goal (± SD)	0.5 (0.6)	0.7 (0.5)	0.7 (0.5)	0.5 (0.3)	0.7 (0.5)	1.4 (1.1)	1.1 (0.9)	0.7 (0.6)	0.6 (0.5)	1.1 (0.9)
% from goal	14	19	19	14	19	36	31	22	19	31

LDL: low-density lipoprotein; HDL: high-density lipoprotein; NAG: not at goal LDL cholesterol.

**Fig. 3.**
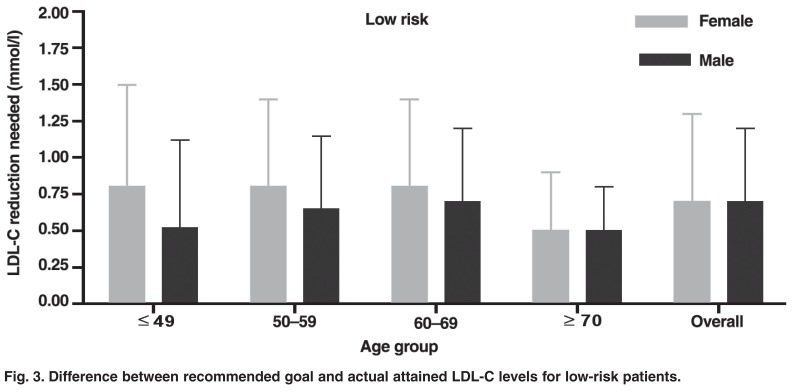
Difference between recommended goal and actual attained LDL-C levels for low-risk patients.

**Fig. 4.**
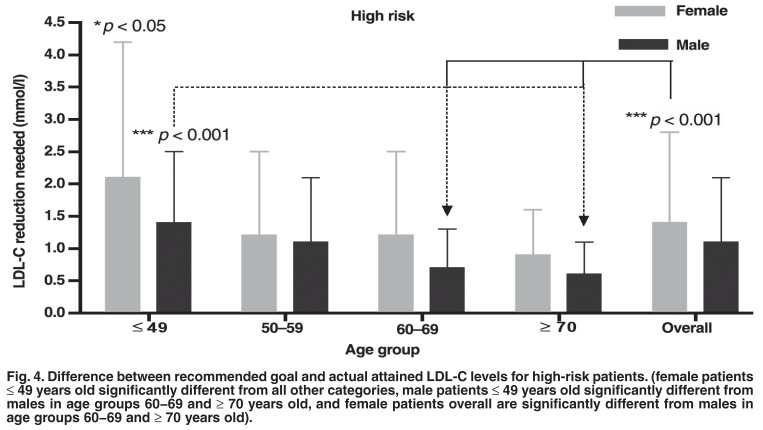
Difference between recommended goal and actual attained LDL-C levels for high-risk patients. (female patients ≤ 49 years old significantly different from all other categories, male patients ≤ 49 years old significantly different from males in age groups 60−69 and ≥ 70 years old, and female patients overall are significantly different from males in age groups 60−69 and ≥ 70 years old).

When separated according to gender, age group and risk category, several further shortcomings of current CVD management were apparent. In the LR group there were no significant differences established between any of the groups analysed for the difference between recommended goal and actual attained LDL-C levels (Fig. 3). High-risk female patients in the ≤ 49-year-old age group had the largest difference between recommended goal and actual attained LDL-C levels compared to other males and females across all age groups (*p* < 0.05) (Fig. 4).

No formal standardised assessment of diet or exercise was evaluated in this study but investigators did report if patients were adhering to any type of diet or exercise therapy. In total, 37.6% (452) of patients indicated they were engaging in exercise therapy [70% (313) not at goal LDL-C] and 55.6% (668) of patients were adhering to some sort of diet therapy [69% (460) not at goal LDL-C].

The primary lipid-lowering drug therapies utilised by the patients were the hydroxymethyl glutaryl coenzyme A reductase inhibitors (statins). Simvastatin (50.3%) and atorvastatin (41.6%) were the statins most frequently prescribed. The most common doses of statin used were simvastatin 10 mg (183 patients), 20 mg (325 patients) or 40 mg (82 patients), atorvastatin 10 mg (316 patients) or 20 mg (123 patients), and fluvastatin 80 mg (34 patients). Only seven patients utilised atorvastatin 80 mg and nine patients used combination therapy of a statin plus a fibrate.

## Secondary goals of treatment

A large proportion of the patient population (53.3%) matched the criteria for the MS. There were 60 (6.3%) non-diabetic and 124 (50%) diabetic patients who had fasting blood glucose levels of > 7 mmol/l. It must be noted that this was a single measurement of fasting blood glucose levels with no HbA_1c_ value measured for non-diabetic patients. A substantial number of diabetic and non-diabetic patients were also not at their BP goals of < 130/80 and < 140/90 mmHg, respectively. In total, 77% (189) of diabetic patients and 32% (303) of non-diabetics were not achieving goal BP levels. Elevated body weight was also highly prevalent in the SA-NAG population with 71.2% of patients being obese or overweight (37.6 and 33.6%, respectively).

## Discussion

As far as we are aware, the SA-NAG survey is the first in South Africa to measure the treatment gap between lipid guidelines and actual goal attainment in dyslipidaemic patients with or without established CVD. Our survey also targeted patients using lipid-lowering medication. The results of this survey indicated that the majority of patients were not achieving newly defined guideline-specified goal levels. In total, 71% of patients were not at goal LDL-C target levels, although it must be noted that the guidelines changed during the study. High-risk patients were less likely to be at goal levels, with 77% of them not at goal, and 63% of LR patients not at goal levels. Those patients who were not at goal were also far from their respective targets − on average, HR patients were 1.1 mmol/l (31%) away from goal, and LR patients were 0.7 mmol/l (19%) away from goal. It is evident from the results of this survey that South Africa’s adoption of the European guidelines for lipid management will place the majority of patients in the category not at goal LDL-C.

Currently, the new recommended goals of therapy are lower than the previously recommended levels. An earlier South African survey in 1993/4, the Cholesterol Monitor survey demonstrated that, overall, 72% of all patients treated in the private sector and 76% of those with established coronary heart disease had total cholesterol levels of > 5.0 mmol/l.26 Taking into account the analysis of the SA-NAG survey under the new guidelines, and the Cholesterol Monitor survey, it is apparent that high proportions of patients in South Africa have suffered from unfavourable lipid levels for an extensive period of time. The situation in South African practice will inevitably become worse as the newer guidelines are expected to have even lower goals of therapy, as new evidence suggests that cholesterol levels lower than current recommendations confer additional benefits.^30^

This study has also identified a certain age group of female patients (< 49 years) who require larger reductions in LDL-C than males to achieve goal levels. Under-treatment and underrecognition of CVD in female patients has been a problem for many years.^19^,^22^,^26^ This could possibly be due to CVD manifesting 10 to 20 years later in female compared to male patients.^31^ In this study, a substantial number of female patients have not been treated to goal, and furthermore, those that are not attaining goal require higher reductions in LDL-C to achieve optimal goal levels. In the United States, more female patients suffer from CVD than male patients.^32^ South Africa does not currently have accurate cause-of-death and disease-incidence statistics but underestimation of the incidence of CVD in female patients will lead to an increased disease burden in the country.^33^

There are several reasons for the shortfall in guideline implementation. International surveys of patients and physicians have identified inadequate titration of doses, patient long-term compliance, time restraints, complexity of guidelines, and finance constraints as the primary reasons for patients not achieving lipid goals.^19^,^25^ The formulation and publication of guidelines are not adequate to drive clinical practice.^34^ Ultimately, education and awareness of guideline recommendations are the most important steps in reducing the lack of adherence to guidelines. Finding innovative strategies to motivate doctors to implement guidelines, and patients to adhere to therapies, as well as discovering efficacious therapies to reduce cholesterol will hopefully make a difference.

This study evaluates the new guideline goals (SA–ESC which will be more difficult to attain in all patients because of new goals of therapy. In our survey, only 45 patients were using the highest doses of statin therapy (atorvastatin 80 mg, simvastatin 80 mg, fluvastatin 80 mg, pravastatin 40 mg), while even fewer patients were on any combination drug therapies. It is evident from the results of this and similar surveys that larger LDL-C reductions than are possible with currently utilised lipid-lowering drug therapies are required for patients to attain goal levels.^22^ Recent trials have clearly demonstrated that more patients achieve guideline-recommended goals with both intensive statin therapy and/or combination drug therapy, and these trials further reiterate the benefits of lowering cholesterol.^4^,^5^,^35^,^36^ In view of these trials, the most logical course for caregivers is to employ a potent statin at the appropriate dose, possibly in combination with another lipid-lowering drug, to maximise LDL-C lowering and thereby obtain a higher rate of success for patients failing to achieve LDL-C goal levels.^22^,^36^

In addition to the lipid goals of treatment, there is a high frequency of other modifiable CVD risk factors, such as elevated blood glucose levels, smoking, elevated BP and obesity. Elevated BP, fasting blood glucose levels and waist circumference, all components of the MS, substantially increase the risk for CVD and diabetes.^16^ Additionally, recent evidence has shown that patients with the MS derived significant benefit from intensive LDL-C lowering with high-dose statin therapy.^37^ The global epidemic of obesity is rife and is clearly highly prevalent in this study group and in South Africa as a whole.^27^ Higher levels of obesity have been shown to increase overall mortality and CVS mortality in particular.^38^ In our survey, an impaired fasting glucose level of > 7 mmol/l was prevalent in 184 patients. Improved monitoring of both diabetic and non-diabetic patients is necessary considering the role of diabetes in CVD.

One major limitation of this study was that we were not able to recruit many black African patients, which could reflect a low awareness of the risk of CVD in this group. It has also been observed that black patients are reluctant to donate blood in surveys because of the negative stigma associated with the HIV/AIDS epidemic, and secrecy regarding HIV status is common.^27^ The incidence of coronary heart disease in black South Africans is on the increase and surveys to quantify the prevalence of this disease are necessary.^8^ This survey also did not assess the number of patients in clinical practice who are dyslipidaemic but undiagnosed. This is also an area of concern, as it has been observed that a large percentage of patients who are dyslipidaemic are undiagnosed and therefore are not treated.^21^,^39^ Standardised assessment of diet and exercise therapies will also improve further studies of this type.

Guidelines are published to present the most effective targets for intervention and the best treatments available to reduce the risk of CVD. Guidelines have established LDL-C goal levels that are practical, attainable levels that will minimise the risk of adverse CVD events. In total, 71% of patients who are using the most efficacious drug therapies available for lipid management are not achieving goal LDL-C levels. The publication of the new guidelines, combined with the results of this survey, necessitates enhanced disease management to reduce the burden of CVDs.
